# Optimal site of pacemaker lead implantation for persistent atrial standstill guided by electroanatomical mapping following a cox-maze procedure: a case report

**DOI:** 10.1093/ehjcr/ytae647

**Published:** 2024-12-02

**Authors:** Sae Ujiro, Soichiro Yamashita, Makoto Takemoto, Masanori Okuda

**Affiliations:** Department of Cardiology, Hyogo Prefectural Awaji Medical Center, 1-1-137 Shioya, Sumoto 656-0021, Japan; Department of Cardiology, Hyogo Prefectural Awaji Medical Center, 1-1-137 Shioya, Sumoto 656-0021, Japan; Department of Cardiology, Hyogo Prefectural Awaji Medical Center, 1-1-137 Shioya, Sumoto 656-0021, Japan; Department of Cardiology, Hyogo Prefectural Awaji Medical Center, 1-1-137 Shioya, Sumoto 656-0021, Japan

**Keywords:** Atrial fibrillation, Electrophysiologic study, Electroanatomical mapping, Pacemaker lead implantation, Case report

## Abstract

**Background:**

Atrial standstill is characterized by the absence of atrial activity. We report a case of a patient with extensive atrial fibrosis who underwent electrophysiologic study (EPS) and electroanatomical mapping (EAM) to identify surviving atrial sites amenable for pacemaker lead implantation.

**Case summary:**

A 72-year-old man with persistent atrial fibrillation (AF) and atrial functional mitral regurgitation/tricuspid regurgitation (MR/TR) underwent a Cox-Maze surgery, mitral and tricuspid valve repair, and biatrial plication. He was referred because of post-operative presyncope symptoms. Electrocardiography revealed atrial standstill and junctional rhythm (JR); however, EAM revealed that both atria were almost entirely scarred and isolated fibrillation in left pulmonary veins and coronary sinus. Junctional rhythm retrogradely conducted around an atrioventricular (AV) node and pacing at this area could conduct to the ventricle through the AV node. An atrial pacing lead was implanted at this area, which yielded a QRS wave similar to the own beat. However, the atrial lead voltage was quite low; hence, ventricular pacing lead was implanted to avoid a future occurrence of pacing failure.

**Discussion:**

This report demonstrates the benefits of EPS and EAM in informing optimal pacemaker implantation for patients with extensive scar in atrium.

**Learning points**

- Electrophysiologic study and electroanatomical mapping were effective in diagnosing paroxysmal arrhythmias and in implanting appropriate pacemaker leads.
- We were able to safely place the right ventricular lead even after the tricuspid valve was plasticated with the ‘clover technique’.

## Introduction

The Cox-maze procedure, developed in the 1980s, is a surgical treatment for atrial fibrillation (AF) that involves creating maze-like incisions and suturing the atrial wall to disrupt abnormal electrical pathways. However, it involves a risk of bradycardia requiring pacemaker implantation, with ≈7.5%–25% of patients requiring pacemaker implantation after the Cox-maze procedure.^1–4^ The ‘clover technique’ is a surgical approach for treating tricuspid regurgitation (TR).^5^ This technique involves suturing the middle of the free end of the tricuspid valve and has yielded good outcomes in patients with TR.^6,7^ There have been no previous reports of right ventricular (RV) lead insertion following the clover technique. We report a case of atrial standstill and atrial tachycardia that developed after the Cox-maze procedure, in which the patient underwent electroanatomical mapping (EAM) and catheter ablation, followed by pacemaker implantation. Right ventricular lead was successfully inserted through the valvuloplasty in clover technique.

## Summary figure

**Figure:**
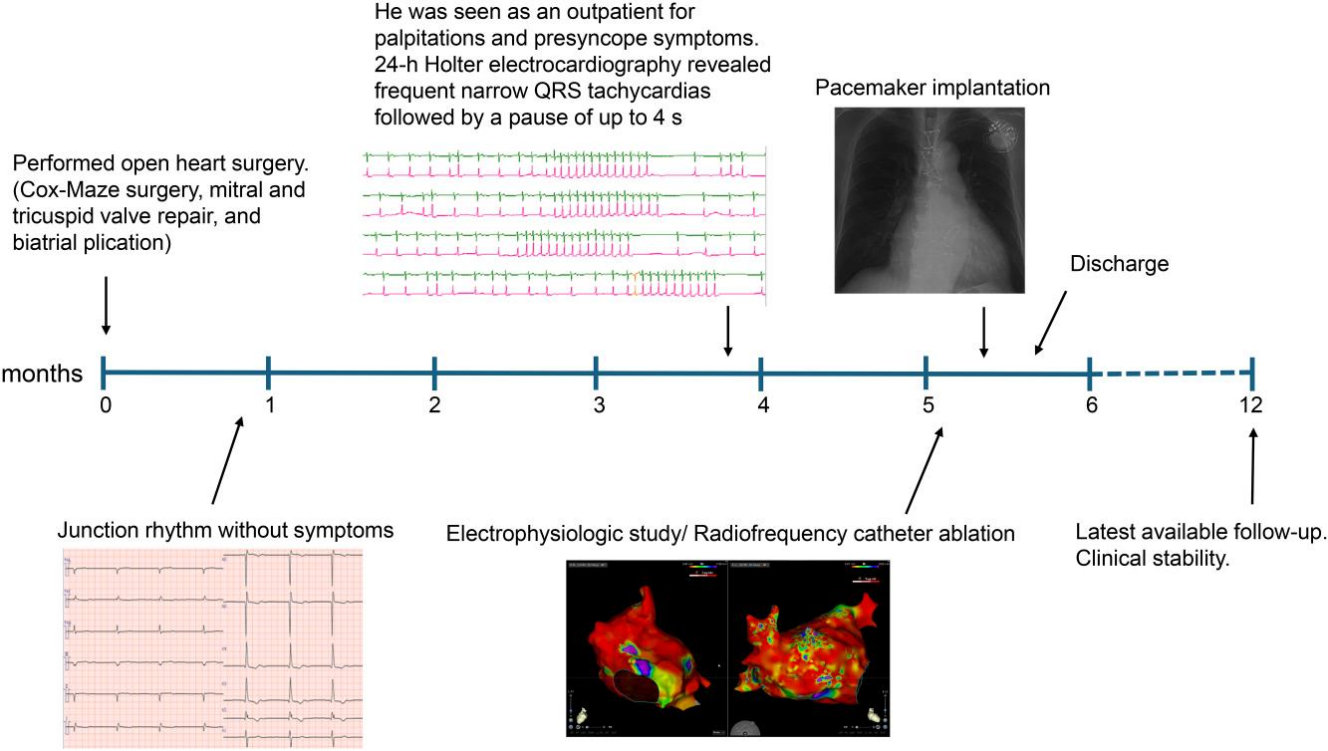


## Case presentation

**Figure 1:**
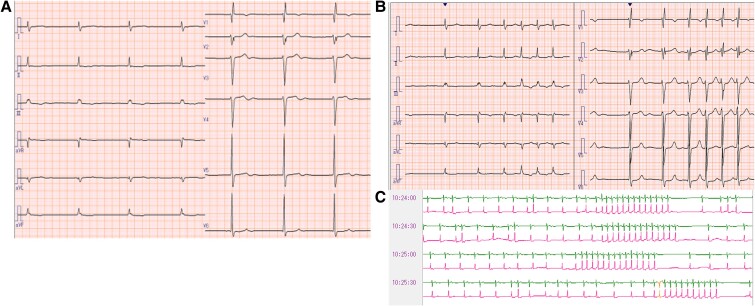
(*A*) A 12-lead electrocardiography after open heart surgery. Atrial standstill and junctional rhythm were observed. (*B*) A 12-lead electrocardiography performed when the patient complained of presyncope symptoms revealed tachycardia with irregular RR intervals. (*C*) A subsequent 24-h Holter electrocardiography revealed frequent narrow QRS tachycardias followed by pauses of ≈4 s throughout the day.

A 72-year-old man had been diagnosed with AF 30 years prior and was being followed up by his primary care physician. He was referred to our hospital due to dyspnoea on exertion. At presentation, a 12-lead electrocardiography (ECG) showed AF rhythm, and transthoracic echocardiography revealed an ejection fraction (EF) of 47% and mildly reduced wall motion. Moreover, there was marked biatrial dilation and the left atrial volume index was 122 mL/m^2^. Additionally, moderate mitral regurgitation (MR) and severe TR associated with annular dilation were observed. Accordingly, the patient was diagnosed with heart failure (HF) due to functional MR/TR associated with persistent AF. He underwent surgical mitral valve plasty, tricuspid valve plasty (TVP), a full Cox-maze procedure, left atrial appendage closure, and biatrial plication (see Supplementary material online, *Figure S1*). Tricuspid valve plasty was performed using the ring plasty and clover technique. Intraoperative myocardial biopsy of the right atrium (RA) revealed wall thinning (thickness = 0.9 mm) and extended fibrosis. Post-operative ECG showed an atrial standstill with a junctional escape rhythm (*Figure 1A*). His hemodynamic state was stable without any symptoms; accordingly, he was discharged. However, at 3 post-operative months, the patient present with palpitation and presyncope symptoms. Subsequent 24-h Holter ECG revealed frequent narrow QRS tachycardias followed by a pause of up to 4 s throughout the day (*Figure 1B* and *C*). Electrophysiologic study (EPS) was performed to diagnose these arrhythmias and inform treatment.

### Electrophysiologic study/radiofrequency catheter ablation

**Figure 2:**
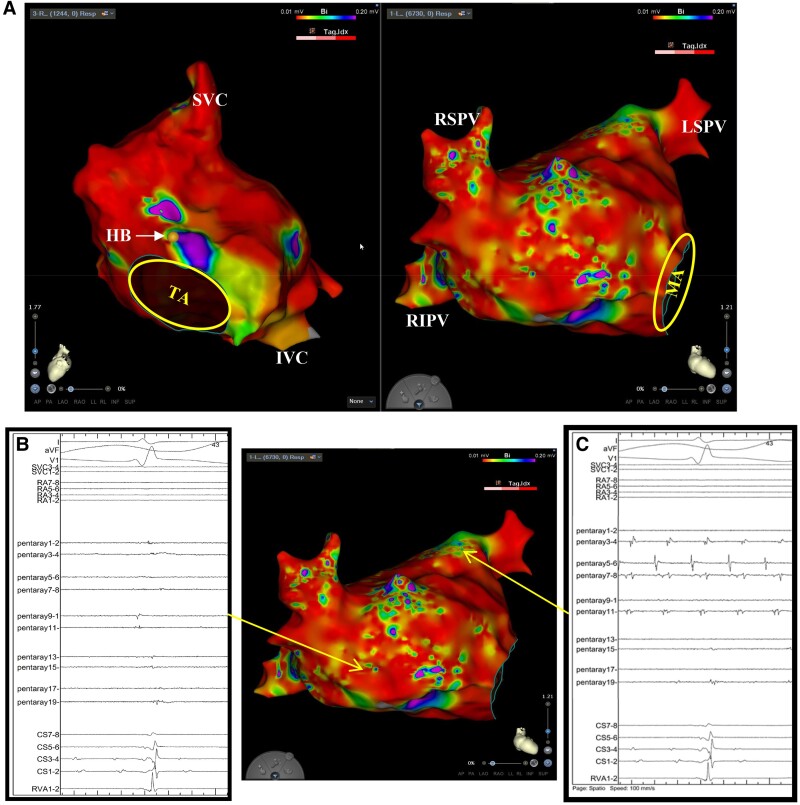
(*A*) Voltage maps revealed widespread biatrial scarring. In the right atrium, potentials were observed exclusively in the His bundle and the surrounding right atrial septum. A multielectrode catheter (Pentaray™) located at the septum of the left atrium showed a retrograde conduction of junctional rhythm (*B*). Electrode catheter located in coronary sinus showed a fibrillation, and there was completely no potential in the right atrium. Atrial fibrillation was isolated in the left pulmonary vein and coronary sinus by the extensive scar (*C*). RA, right atrium; LA, left atrium; HB, His bundle; IVC, inferior vena cava; SVC, superior vena cava; RSPV, right superior pulmonary vein; LSPV, left superior pulmonary vein; RIPV, right inferior pulmonary vein; MA, mitral annulus; TA, tricuspid annulus; CS, coronary sinus.

**Figure 3:**
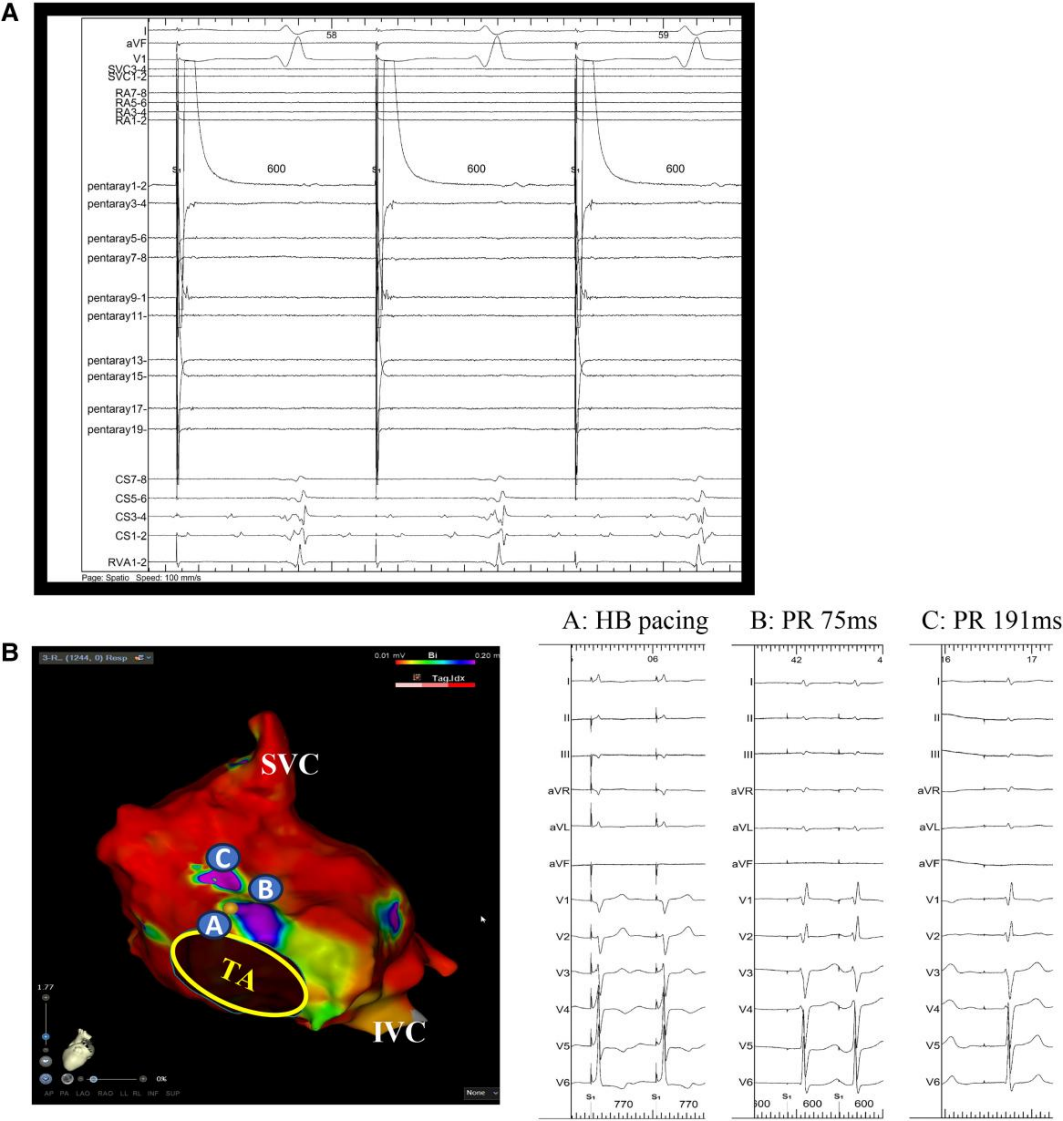
(*A*) Tracing of the pacing from the left atrial septum. Pacing could conduct to the ventricle through the atrioventricular node. (*B*) The PR interval paced by each location. Tracing A showed an electrocardiogram with the His-bundle pacing. Pacing from the right atrial septum conducted to the ventricle with different PR intervals (*B* and *C*).

**Figure 4:**
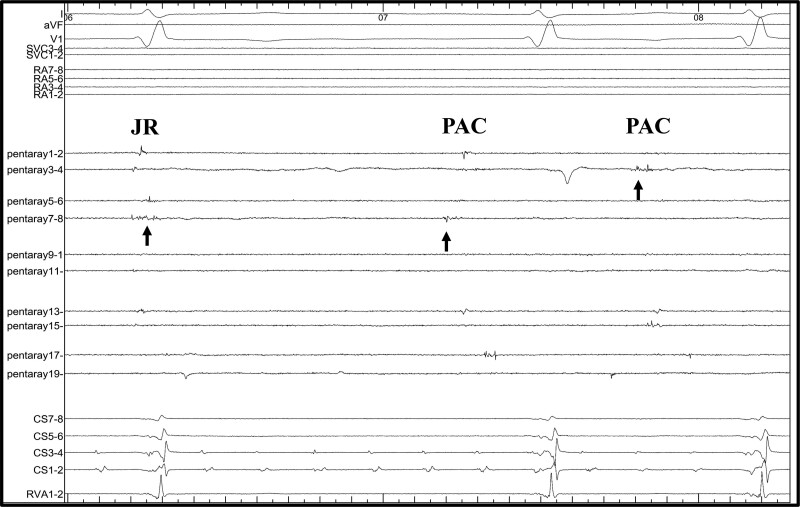
A tracing when atrial tachycardia was started. Multielectrode catheter was placed at the left atrial septum. The first beat with late potentials was a retrograde conduction from the junctional beat. The second and third beats firing from the multiple origins of the left atrial septum were documented. JR, junctional rhythm; PAC, premature atrial contraction.

Electrophysiologic study was performed under general anaesthesia. The femoral vein was percutaneously cannulated under local anaesthesia induced by 1.0% lidocaine. After atrial septal puncturing, a voltage mapping was created by 20-pole electrode catheter (PENTARAY, Biosense Webster, CA, USA), which revealed an extensive scar spreading in the left atrium (LA; *Figure 2A*). Given the atrial scarring, AF was isolated within left pulmonary veins and coronary sinus (CS; *Figure 2B* and *C*). Moreover, there was retrograde conduction of junctional rhythm at the LA septum. When atrial pacing was performed at the LA septum, they conducted through the atrioventricular (AV) node to the ventricles (*Figure 3A*). Electroanatomical mapping of the RA revealed extensive scarring with a localized AF rhythm only in the CS. Similar to in the LA, retrograde conduction of the junctional rhythm was only observed around the His bundle, with pacing from this area conducting them to the AV node (*Figure 3B*). The His-ventricular time was 42 milliseconds, which indicated no conduction disturbance below the His bundle. These results indicated that atrial pacing around the AV node could be achieved without affecting the post-operative tricuspid valve repair. Regular R-R, narrow QRS, tachycardia was caused by paroxysmal atrial tachycardias (PATs) from the LA septum (*Figure 4*). Specifically, PATs were originated from various sites of the border zone between the LA septum and scar. Accordingly, we extensively mapped and ablated them.

Intracardiac echocardiography of the tricuspid valve revealed that the valve ring was pulled together by the ring and coaptation had been partially achieved (see Supplementary material online, *Figure S2*). The septal leaflet was freely movable, which suggested that the clover technique stitch may have been removed. Nevertheless, TR was controlled to a mild degree (see Supplementary material online, *Figure S3*). Accordingly, we initially decided to use atrial pacing alone without a ventricular lead in order to avoid lead-induced TR. Although the conduction system pacing was considered, especially for the clover technique, there was a possibility of compressing and destroying the sutured valve by a stiff guiding sheath for conduction system pacing-lead placement.

### Pacemaker implantation

**Figure 5:**
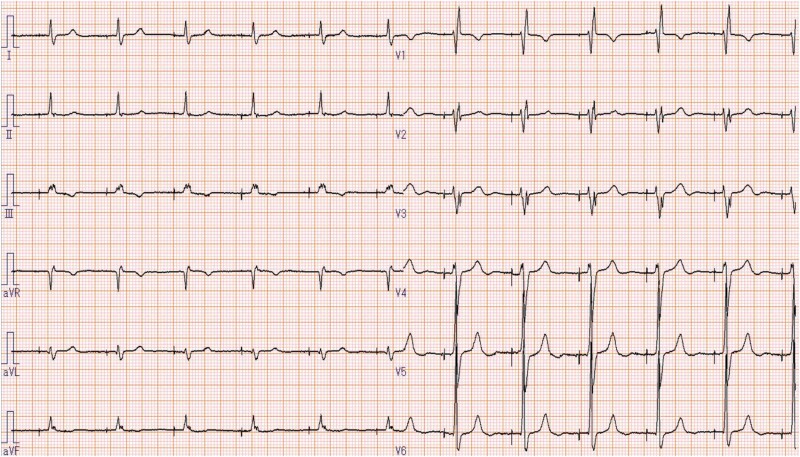
A final 12-lead electrocardiography.

The left subclavian was locally anaesthetized with 1.0% lidocaine; subsequently, an atrial lead was transvenously inserted through the left subclavian vein. Next, the atrial lead was placed and screwed around the AV node, which had been confirmed as the suitable site for pacing by the previously performed EPS. The atrial pacing threshold was fine; however, the atrial voltage was very low at 0.3 mV. Therefore, we performed ventricular lead implantation to the high septum of the RV. Implantation was successfully completed without any complications. A pacemaker check performed after 1 week was uneventful and the patient was discharged with an atrial-inhibited (AAI) mode of 70 bpm setting (*Figure 5*). There has been no recurrence of atrial tachycardia and syncope for >2 years.

## Discussion

This article describes a case of symptomatic bradycardia that developed after cardiac surgery, in which an appropriate pacemaker implantation was informed by EAM. Electrocardiography showed a junctional rhythm without any atrial wave, which mimicked an atrial standstill. Electroanatomical mapping revealed that the area of electrical conduction to the AV node was limited to a septal portion. Further, fibrillation was isolated in the pulmonary veins and CS due to the Cox-Maze procedure, bi-atrial plication, and a natural scar resulting from atrial remodelling. Therefore, EAM was crucial for determining the appropriate site of an atrial pacing lead. Electroanatomical mapping has been previously shown to inform appropriate repositioning of atrial pacing leads following failed replacement in four cases.^8^ Moreover, Aronis *et al*. published a case report of atrial standstill similar to our case.^9^ In this previous report, EAM of the RA revealed an isolated island of surviving atrial tissue. Although pacing at this identified site could have resulted in a local capture, it did not yield ventricular activation due to intra-atrial conduction block. Accordingly, they abandoned atrial lead placement and changed the strategy to single-chamber left bundle pacing. Taken together, these findings indicate that EAM-guided pacemaker implantation may be effective in cases such as the present case, where there is extensive atrial fibrosis.

Although there have been no other reported cases of RV lead placement after the clover technique, as in the present case, there have been few reports of RV lead insertion after TVP.^10^ A retrospective study comparing groups with and without RV lead insertion after TVP reported that RV lead implantation was a risk factor for TR recurrence and poor prognosis.^11^ However, another retrospective study reported no significant difference in the incidence of TR between groups with and without RV lead insertion after TVP.^12^ Therefore, further research is warranted.

## Conclusion

This article describes a case in which EPS and EAM informed secure placement of active fixation endocardial pacing leads in the RA.

## Supplementary Material

ytae647_Supplementary_Data

## Data Availability

The authors confirm that written consent for submission and publication of this case report including the images and associated text have been obtained from the patient in line with COPE guidance.

## Lead author biography

Senior resident at Hyogo Prefectural Awaji Medical Center.

## Notes

**Consent:** The authors confirm that written consent for submission and publication of this case has been obtained from the patient in line with the Committee on Publication Ethics (COPE) guidelines.

**Funding:** None declared.
