# Supplementary material for: Optimal site of pacemaker lead implantation for persistent atrial standstill guided by electroanatomical mapping following a cox-maze procedure: a case report
Source: Eur Heart J Case Rep. 2024 Dec 2;8(12):ytae647. doi: 10.1093/ehjcr/ytae647 (PMC11647587; doi:10.1093/ehjcr/ytae647)
Supplement: ytae647_Supplementary_Data [file ytae647_supplementary_data.zip › supplemental data.pptx]

## Slide 1
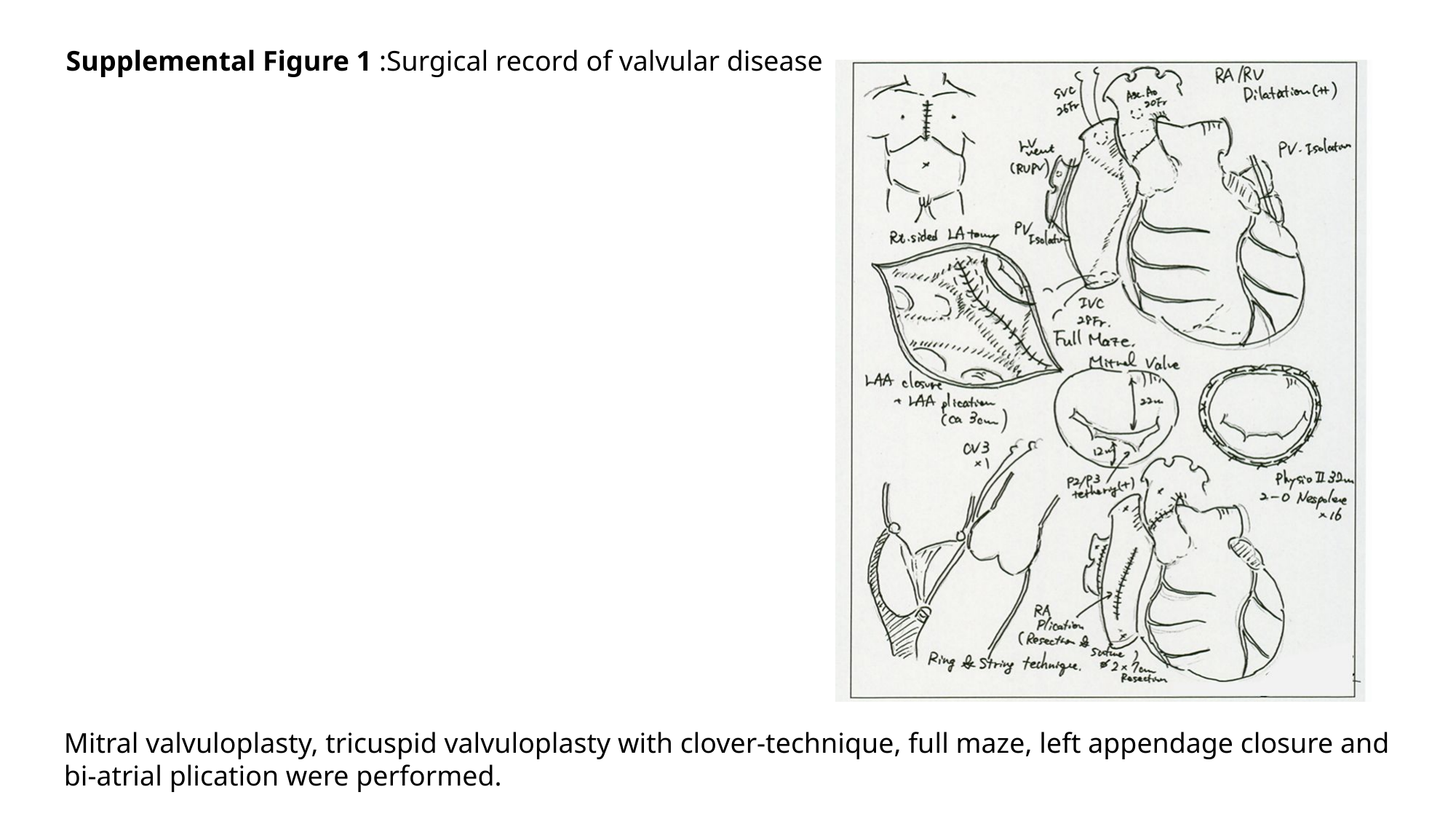

Supplemental Figure 1 :Surgical record of valvular disease
Mitral valvuloplasty, tricuspid valvuloplasty with clover-technique, full maze, left appendage closure and bi-atrial plication were performed.

## Slide 2
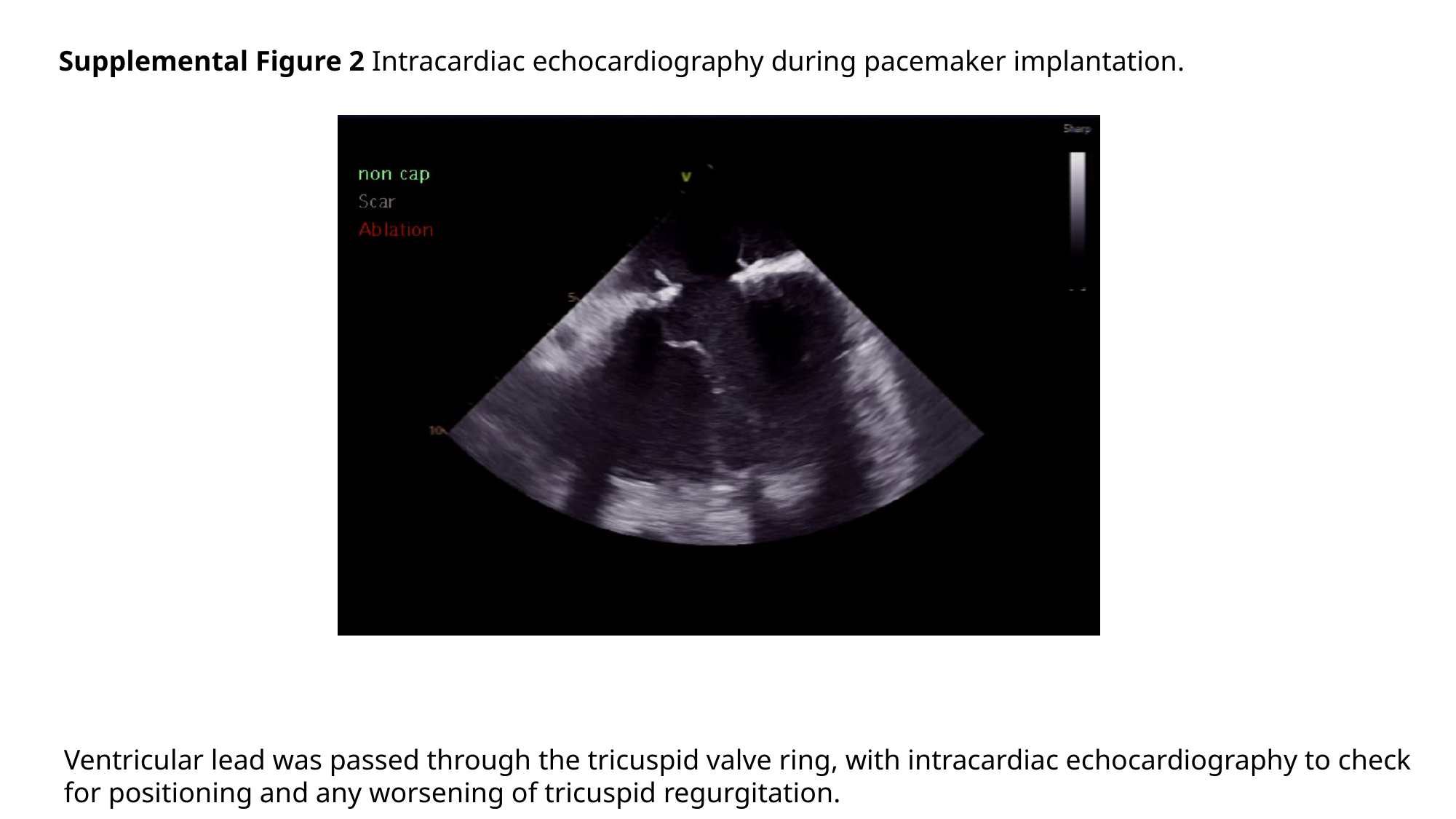

Supplemental Figure 2 Intracardiac echocardiography during pacemaker implantation.
Ventricular lead was passed through the tricuspid valve ring, with intracardiac echocardiography to check for positioning and any worsening of tricuspid regurgitation.

## Slide 3
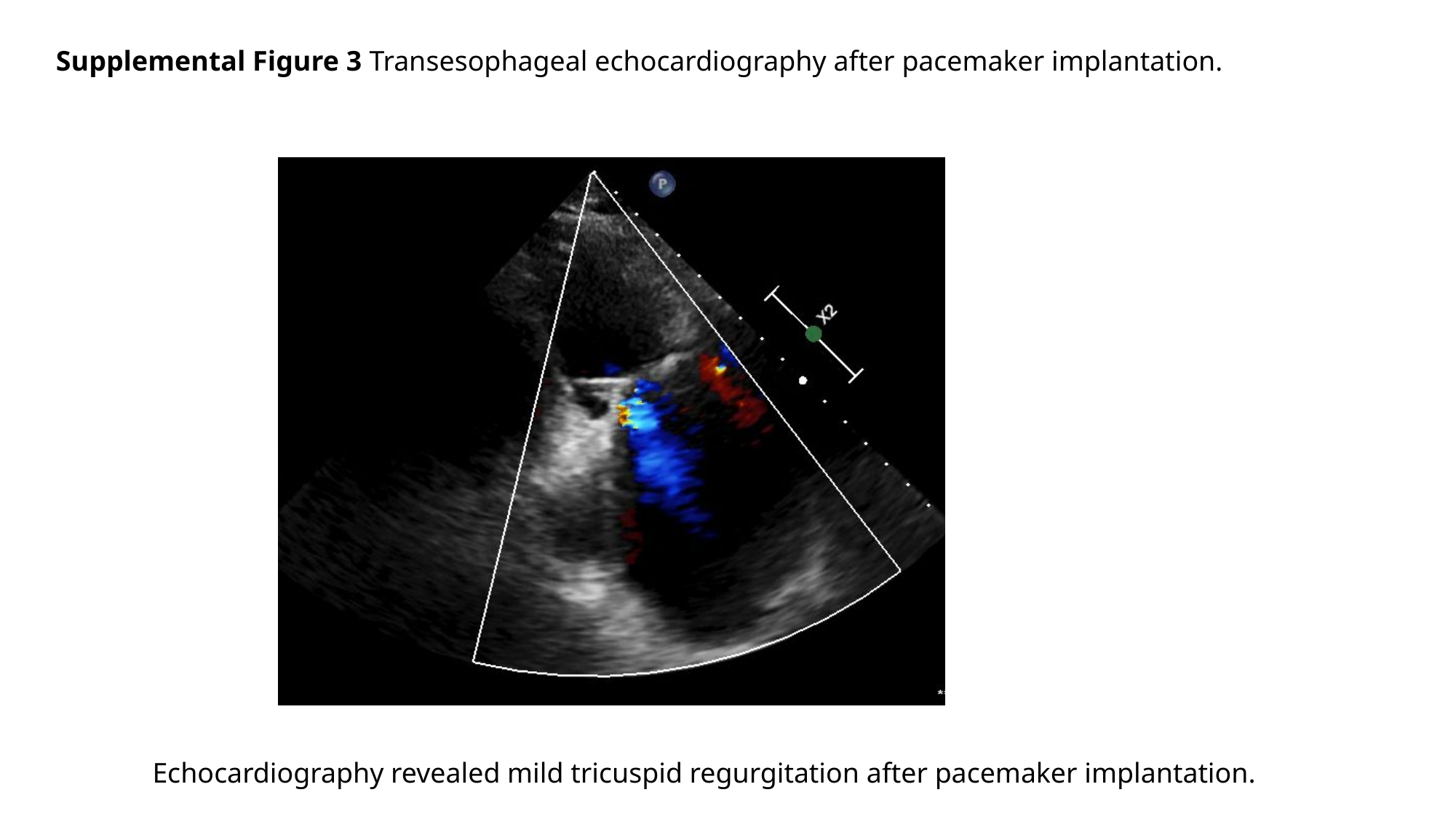

Supplemental Figure 3 Transesophageal echocardiography after pacemaker implantation.
Echocardiography revealed mild tricuspid regurgitation after pacemaker implantation.
